# Development in Detection Methods for the Expression of Surface-Displayed Proteins

**DOI:** 10.3389/fmicb.2022.899578

**Published:** 2022-04-25

**Authors:** Chenglong Ma, Chunyang Jiang, Dongping Zhao, Shuhao Li, Ronggui Li, Lei Li

**Affiliations:** ^1^College of Life Sciences, Qingdao University, Qingdao, China; ^2^School of Basic Medicine, Qingdao Medical College, Qingdao University, Qingdao, China

**Keywords:** ELISA, molecular display platform, phage display, SH2, chromodomain

## Abstract

Directed evolution is a widely-used engineering strategy for improving the stabilities or biochemical functions of proteins by repeated rounds of mutation and selection. A protein of interest is selected as the template and expressed on a molecular display platform such as a bacteriophage for engineering. Initially, the surface-displayed protein template needs to be checked against the desired target *via* ELISA to examine whether the functions of the displayed template remain intact. The ELISA signal is subject to the protein-target binding affinity. A low-affinity results in a weak ELISA signal which makes it difficult to determine whether the weak signal is because of low affinity or because of poor expression of the protein. Using a methyllysine-binding chromodomain protein Cbx1 that weakly binds to the histone H3K9me3 peptide, we developed and compared three different approaches to increase the signal-to-background ratio of ELISA measurements. We observed that the specific peptide-binding signal was enhanced by increasing the Cbx1 phage concentration on the ELISA plate. The introduction of previously known gain-of-function mutations to the Cbx1 protein significantly increased the ELISA signals. Moreover, we demonstrated that the H3K9me3-specific binding signal was enhanced by fusing Cbx1 with a high-affinity phosphotyrosine-binding protein and by coating the ELISA plate with a mixture of H3K9me3 and phosphotyrosine peptides. This approach also worked with binding to a lower affinity momomethyllysine peptide H3K9me1. These approaches may help improve ELISA experiments when dealing with low-affinity ligand-protein interactions.

## Introduction

Protein engineering through directed evolution is an effective way to obtain proteins with enhanced properties [e.g., increased binding affinity (Nowak et al., 2020) and catalytic activity (Bilal et al., 2018)], which has significantly impacted biological research and biotechnology. Generally, the protein of interest is selected as the template and is engineered by applying random or site-directed mutagenesis techniques to generate a library of protein variants. This library is then screened to identify the mutations that confer the desired property. Several molecular display platforms have been developed, on which the engineered variants are tethered to the surface of phage (Smith, 1985), bacteria (Francisco et al., 1993), or yeast (Boder and Wittrup, 1997) cells. In the cell surface display, each cell is transformed with a single vector encoding a protein variant fused to a cell-surface anchor protein and therefore, the variant is accessible to the extracellular space. The surface-displayed proteins include antibody derivatives [e.g., scFv (Tadayoni et al., 2021) and Fab (Smith and Kaiser, 2016)] and some proteins that bind to specific targets. For example, ADP-ribose binding macro domain protein Af1521 was engineered with a 1,000-fold increased affinity towards ADP-ribose (Nowak et al., 2020), the Fyn SH2 domain was engineered with over 100-fold increased affinity towards phosphotyrosine (pY; Kaneko et al., 2012; Li et al., 2021), and H3K9me3-binding protein Cbx1 was engineered to specifically recognize H3K9me3 with enhanced affinity (Hard et al., 2018; Albanese et al., 2020).

Protein engineering through directed evolution starts with the premise that the protein template is functional and well displayed on the surface. That is necessary; otherwise, the variants in the library constructed based on the template may be poorly expressed, and those variants with the desired property may not be successfully selected from the library. For instance, before the Fyn SH2 domain was considered a template to find variants with potentially higher affinity to phosphotyrosine, it should be ensured that this SH2 domain is displayed on the platform surface with the original phosphotyrosine-binding property. When conducting directed evolution experiments by the phage display technology, the expression and function of the protein presented on the phage surface need to be routinely checked against the desired target *via* phage ELISA, in which phage-containing supernatants were commonly used. However, the phage ELISA signal is subject to a few factors, such as buffer composition and the protein-target binding affinity. A low binding affinity may result in a weak ELISA signal, making it difficult to determine whether the weak ELISA signal is caused by poor protein expression on the phage or by low-affinity binding of the variant to the target. This study investigated phage-displayed proteins with low affinities to their targets and figured out how to check whether the proteins are well expressed on the phage surface. Here, we expressed as study cases the phage-displayed human chromodomain protein Cbx1, which recognizes Histone 3 lysine 9 (H3K9) mono-, di- and tri-methylation (Liu et al., 2010), as well as human Fyn SH2 domain, which recognizes tyrosine phosphorylation (Kaneko et al., 2012; Li et al., 2021).

## Materials and Methods

### Bacterial Strains and Plasmids

The *E. coli* XL1-blue was purchased from New England Biolabs, and *E. coli* CJ236 was from Takara Biotechnology. The phagemid pFN-OM6 was obtained from Shanghai AsiaUnited Antibody Medical Co., Ltd. The Fyn SH2 phagemid contained N-terminal Flag-tagged Fyn SH2 domain (Kaneko et al., 2012; Li et al., 2021), and the Cbx1 phagemid included N-terminal Flag-tagged Cbx1 protein (UniProt ID: P83916, 1–185). The Fyn SH2-Cbx1 fusion phagemid was constructed by connecting N-terminal Flag-tagged Fyn SH2, a 3x GGGGS linker and the Cbx1 protein.

### Reagents

Tryptone, Yeast Extract were purchased from Thermo Fisher Scientific, OXOID. Agar powder and skim milk powder was purchased from the Solarbio company. Glycerol, Sodium chloride (NaCl), Sodium hydroxide (NaOH), and other reagents of analytical reagent grade were purchased from Sinopharm Chemical Reagent (Shanghai, China); T4 Polynucleotide Kinase, T4 DNA Polymerase and T4 DNA Ligase were purchased from New England Biolabs (NEB).

### Media for Bacterial Growth

#### 2 × Yeast Extract Tryptone (YT) Media

Five grams of NaCl, 10 g Yeast Extract and 16 g Tryptone were dissolved in 900 ml ddH_2_O. The pH of 2YT mediun was adjusted to 7.5 with about 1 ml 4 M NaOH and the volume of medium was made up to 1 l with ddH_2_O. Then the medium was sterilized in an autoclave and cooled to room temperature for use.

#### Luria-Bertani (LB) Medium Plate

Ten grams per litre of Tryptone, 5 g/l Yeast Extract, 10 g/l NaCl, 15 g/l agar powder, adjusted to a pH of 7.5 with 4 M NaOH were sterilized in an autoclave. The medium was allowed to cool to 50°C, after which carbenicillin (final concentration 50 μg/ml) was added. The medium was poured in to the plates in the super clean bench. Then the LB medium plates were cooled to room temperature for use.

#### Phage Preparation and Precipitation of Phage Particles

From a fresh LB/carb plate, a single colony of *E. coli* XL1-blue [New England Biolabs (NEB)] harbouring the phagemid pFN-OM6 was picked into 2 ml of 2YT media supplemented with Carb (carbenicillin, 50 μg/ml). The colony was incubated with shaking at 200 rpm at 37°C for 6 h, and M13KO7 helper phage was added with a final concentration of 1 × 10^10^ pfu/mL. After the incubation with shaking at 200 rpm at 37°C for 45 min, the culture was transferred to a 250 ml flask that contained 50 ml 2YT media supplemented with Carb (carbenicillin, 50 μg/ml) and Kana (kanamycin, 25 μg/ml). The flask was incubated on a shaking platform with a speed of 200 rpm at 37°C for 16 to 18 h. The culture was centrifuged for 10 min at 16,000 g at 4°C, and the amplified phages were solved in the supernatant.

The phage supernatant was transferred to a new tube containing 1/5 volume of PEG8000/NaCl and incubated for 30 min at an ice bath to concentrate bacteriophages. After 10-min centrifugation at 16,000 g at 4°C, the supernatant was discarded. The phage pellets were resuspended in 1 ml of 1×PBS and transferred to a 1.5-mL microcentrifuge tube. After 10-min centrifugation at 16,000 g at 4°C, the supernatant was transferred to a new 1.5-mL microcentrifuge tube.

#### Phage ELISA

In a 96-well microplate (Costar, No. 42592), 6 pmol streptavidin (Solarbio, S9171) was coated per well in 50 μl 1×PBS at 4°C overnight. The 3% (m/v) skim milk was prepared by dissolving skim milk powder (Solarbio, No. D8340) in PBS and could be regard as blank control. Next, the solution in the well was discarded, and 200 μl/well 3% (m/v) skim milk was added for blocking at room temperature for 1 h. After removing the solution, the wells were washed three times by the PT buffer (PBS supplemented with 0.05% Tween 20, Solarbio, T8220). Then 50 μl biotinylated peptides (24 pmol/well) were added for immobilization for one hour at room temperature. For the group of blank control, 50 μl 3% (m/v) skim milk were added rather than biotinylated peptides. After washing by the PT buffer three times, the phage solution was added for binding for one hour at room temperature. Unbound phages were washed away six times by the PT buffer. 50 μl anti-M13/HRP antibodies (Sino Biological, No. 11973) were added and incubated for 30 min at room temperature. The wells were washed six times by the PT buffer and two times by the PBS buffer. 50 μl freshly prepared TMB substrates (Innoreagents, TMB-S-003) were added to develop, and 50 μl 1.0 M H_3_PO_4_ were added to stop the reaction. The signals were read spectrophotometrically at 450 nm in a plate reader.

#### Cbx1 Mutants Construction Based on Kunkel Method

The dut^−^/ung^−^ *E. coli* CJ236 (Takara Biotechnology, Dalian, China) was applied to produce the uracil-containing ssDNA (dU-ssDNA), as it lacks dUTPase and uracil-N glycosylase activities, and the dut^+^/ung^+^ *E. coli* XL1-blue (New England Biolabs) was applied to proliferate newly synthesized strands and digest the uracil-containing parental template. Primer 1 (TATCTTCTAAAGTGGGCAGGTTTCTCAGATGAG) and primer 2 (CCAGAAGAAAATCTGTTCTGCCCTGACCTTATT) were used in a combinatorial mutation to construct Cbx1 double mutant (K43A/D59F). Primer 3 (GAGGAAGAGGAATATGAAGTGGAAG AAGTTCTTGATCGGCGA) and primer 4 (CCAGAAGAAAATCTGTCTTGCCCTGACCTTATT) were used to construct Cbx1 triple mutant (V22E/K25E/D59S).

#### Production of dU-ssDNA

The dU-ssDNA of the wild-type human Cbx1 as the template in the Kunkel reaction was made as described before (Tonikian et al., 2007). The phagemid Cbx1-pFN-OM6 was transformed into the CJ236 cells. The following day, a single clone was inoculated in 2 ml 2 × YT medium containing carbenicillin (50 μg/ml) at 37°C, shaking at 200 rpm for 5 h. M13K07 helper phage (New England Biolabs, Ipswich, MA, United States) was then added into the medium with a final concentration of 1 × 10^10^ pfu/mL, and the cells were incubated for another 1 h. The whole culture was transferred into 100 ml 2 × YT/Carb (50 μg/ml)/Kana (25 μg/ml)/Uridine (0.25 μg/ml) medium, incubated at 37°C and shaken at 200 rpm for 18 to 20 h. The culture was then centrifuged at 16,000 g for 10 min to remove the cells. Phage particles in the supernatant were precipitated with PEG8000/NaCl and were resuspended in 1 ml phosphate-buffered saline (PBS). The phage dU-ssDNA was extracted by the Spin M13 kit (Omega Bio-Tek, Norcross, GA, United States), quantified by Nanodrop1000 spectrophotometer, and analyzed by agarose gel electrophoresis.

#### *In vitro* CCC-dsDNA Synthesis

A classic three-step procedure of the Kunkel method was employed to synthesize heteroduplex covalently closed, circular, double-stranded DNA (CCC-dsDNA; Tonikian et al., 2007). Briefly, 20 pmol mutagenic oligonucleotides were phosphorylated in a 20 μl reaction with T4 polynucleotide kinase at 37°C for 1 h. Then, the product was annealed with 2 pmol dU-ssDNA by using cooling programs. Finally, the CCC-dsDNA was synthesized by a fill-in reaction with T4 DNA polymerase and T4 DNA ligase at 22°C overnight.

## Results

### The Phage ELISA Signal for Cbx1 and H3K9me3 Interaction Is as Weak as the Background Signal

To investigate differences between low affinity and high-affinity interactions in phage-ELISA experiments, we chose two protein domains, the chromodomain protein Cbx1 and the tyrosine kinase Fyn SH2 domain, representing low- or high-affinity interactions. Human Cbx1 protein binds to the histone 3 lysine 9 trimethylated peptide (H3K9me3: KQTARKme3STGGKA) with relatively low affinity (Li et al., 2021), measured by fluorescence polarization, compared with the interaction between the Fyn SH2 domain and the MidT-pY^324^ peptide (EPQpYEEIPIYL; Kaneko et al., 2012). The dissociation constant value (K*_d_* = 3.2 μM) of the former is ten times larger than that (K*_d_* = 0.327 μM) for the latter (Li et al., 2021). Additionally, Cbx1 does not bind to the corresponding nonmethylated H3K9 peptide, and the Fyn SH2 domain fails to bind to the nonphosphorylated MidT-Y^324^ peptide (Kaneko et al., 2012; Li et al., 2021).

**Figure 1:**
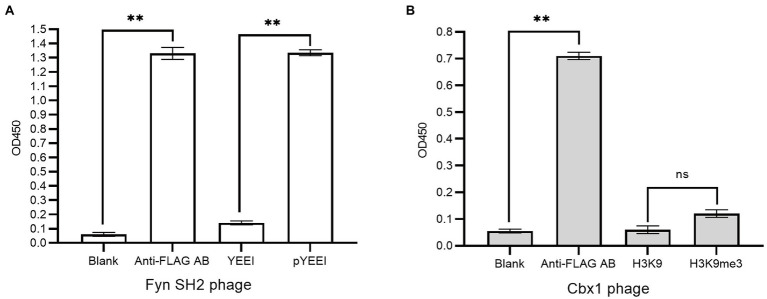
Supernatant phage ELISA showed the expression level and binding to the pY (MidT-pY324) peptide of the Fyn SH2 domain **(A)** and the H3K9me3 peptide of Cbx1 **(B)**, respectively. ELISA signals (OD450) are plotted as a function of the supernatant phages added to the streptavidin-coated wells conjugated with biotinylated modified peptides (pY or H3K9me3), unmodified counterparts (Y or H3K9) as negative controls and skim milk as the blank control, respectively. The anti-FLAG antibody (AB)-coated wells were taken as positive controls. The experiments were repeated three times and a statistical significance in comparison was calculated using unpaired two-sample t-test: ns: not significant; ^*^p < 0.05; ^**^p < 0.01. The same statistical analysis was performed in the following figures.

We fused flag-tagged Cbx1 and Fyn SH2 domain to the capsid protein P3 of the phagemid pFN-OM6, respectively, and checked their ligand binding by measuring their interactions with the corresponding biotinylated targets using supernatant phage ELISA. The streptavidin-coated wells were used to measure the protein and target interactions, taking the biotinylated unmodified targets as the negative control and milk as the background. The anti-FLAG antibody-coated wells were applied as the positive control to check the expression of the engineered P3. We considered an ELISA signal as positive when the signal intensity is averagely triple as strong as the background intensity, and they are statistically different from each other with a value of *p* <0.05. Figure 1 showed that positive controls for both phages had stronger signals (1.37 or 0.71) than the background signals (0.05), indicating that the engineered P3 was well expressed. Fyn SH2 domain had a strong ELISA signal (1.31) towards the pY peptide but a weak signal (0.14) towards the negative control, indicating that Fyn SH2 was functionally displayed (Figure 1A). By contrast, the ELISA signal (0.11) of Cbx1 and the H3K9me3 peptide is close to the negative control (0.06; value of *p* = 0.057), showing the uncertainty about whether Cbx1 was well or poorly expressed. This low ELISA signal of the Cbx1-H3K9me3 complex might be due to either their low affinity or the incorrect design of the CBX1-P3 fusion gene. We figured it out by performing several different approaches, each with its advantages and disadvantages.

### Approach One: High Phage Concentration Increases the ELISA Signal

**Figure 2:**
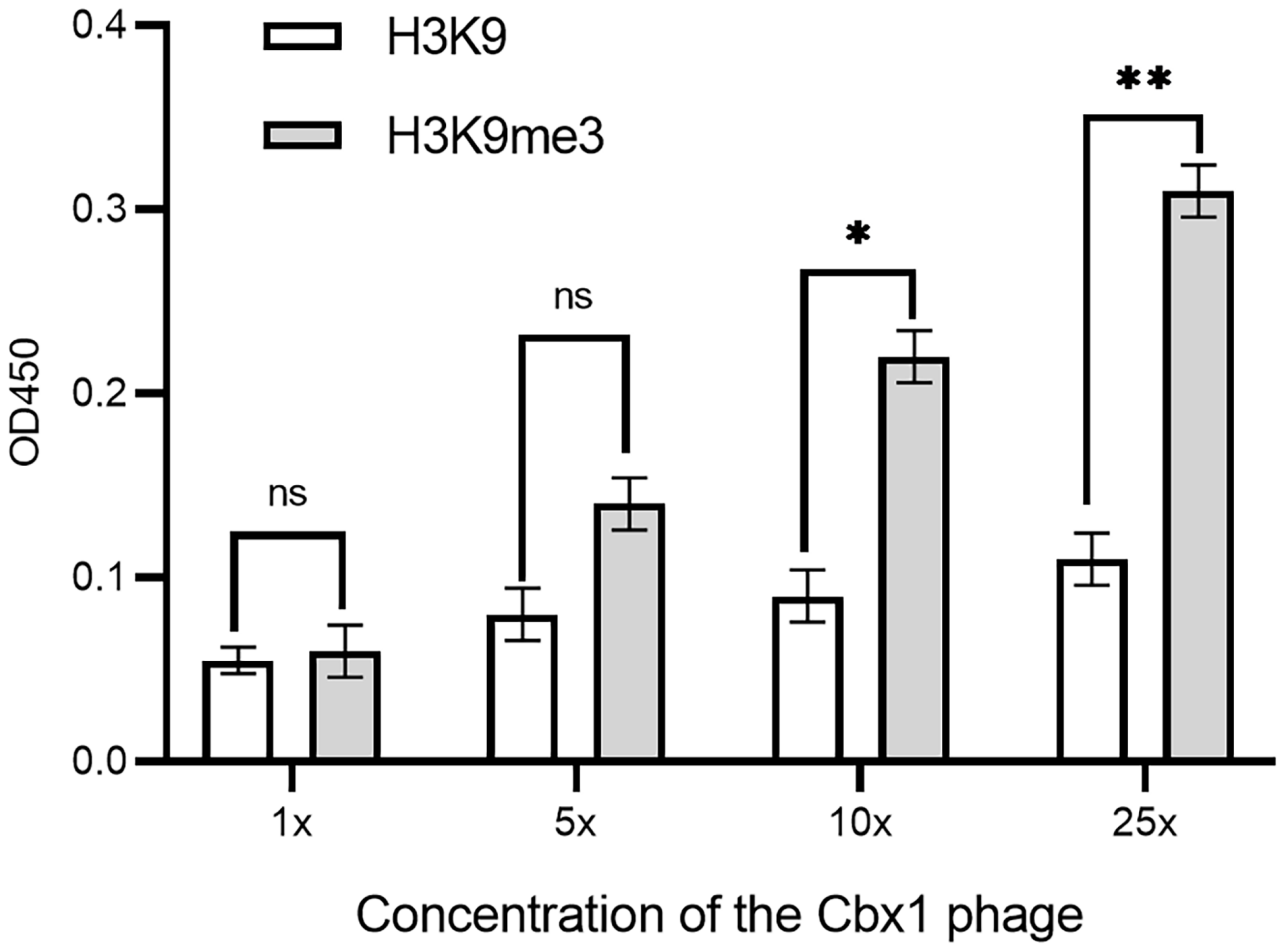
ELISA signals were plotted as a function of the Cbx1-containing phage with different phage concentrations added to the streptavidin-coated wells conjugated with biotinylated H3K9me3 or H3K9 peptides as the negative control. 1x concentration represents a solution with a phage concentration similar to the supernatant phage. The experiments were repeated three times.

As there is a relatively low concentration of phage particles in the supernatants, it is easy to elute the bound phages if the phage-displayed protein has a low affinity to the target. These may result in a low ELISA signal for the Cbx1 and H3K9me3 binding. One possible solution is the increase in the number of bound phages. To this end, we concentrated the phage particles in the supernatants and repeated the phage ELISA. Figure 2 shows that the ELISA signal for the Cbx1 and H3K9me3 complex is proportional to the phage concentrations. The ELISA signal (0.31) related to the highest phage concentration is five times as strong as that (0.06) of the supernatant phage and triple that (0.11) of its binding to the H3K9 peptide (as negative control). These observations indicate that Cbx1 is functionally displayed on the phage surface. Moreover, they suggest that phage concentration increases the phage amount and more bound phages remain after elution, increasing the ELISA signal. Nevertheless, the ELISA signal for the negative control increased from 0.05 to 0.11 with the concentrations, possibly caused by the increase of nonspecific binding. This approach may be unsuitable for testing the protein with much low binding affinity to its target.

### Approach Two: Gain-of-Function Protein Mutations Boost the ELISA Signal

**Figure 3:**
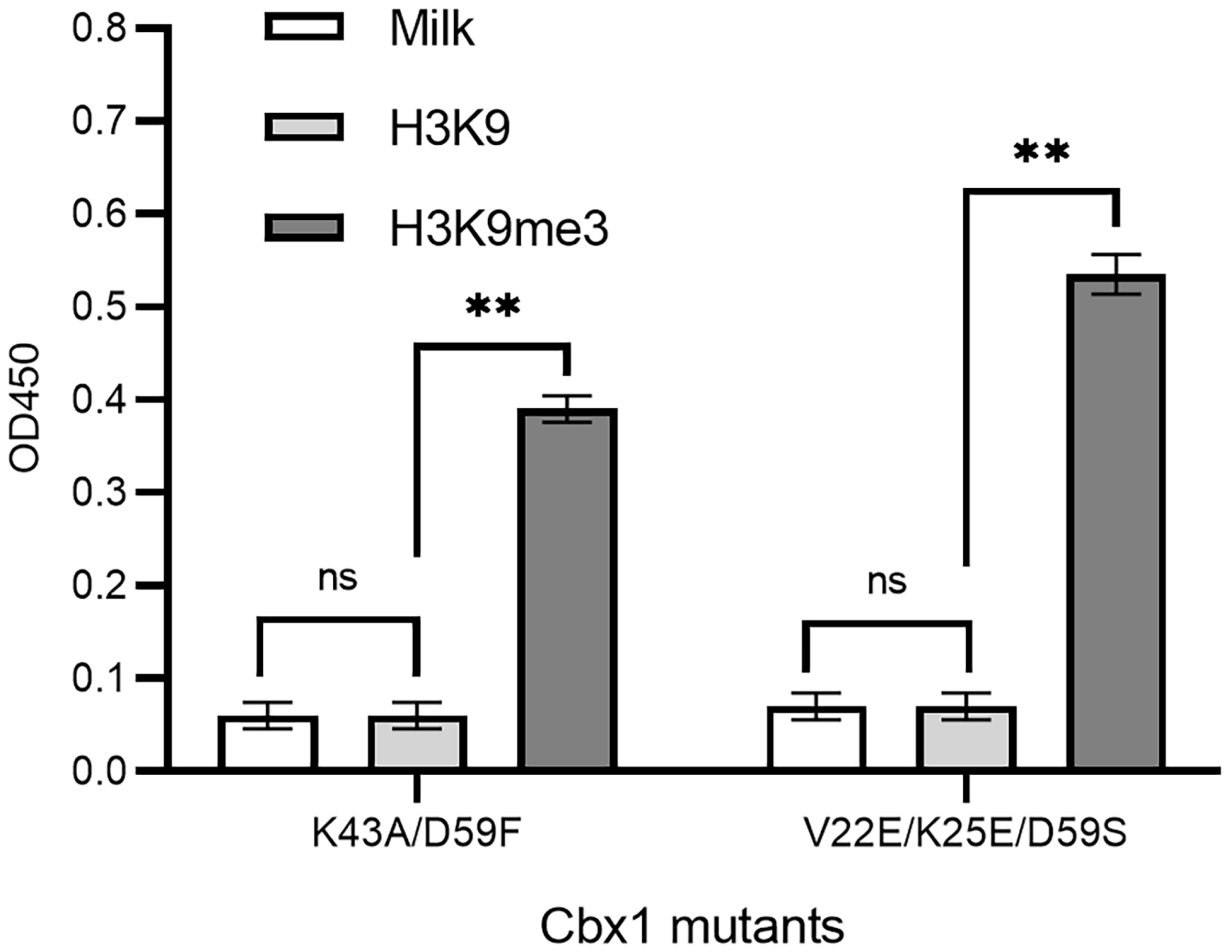
Supernatant phage ELISA showed the expression level and binding to the H3K9me3 peptide of the Cbx1 mutants. The milk and the H3K9 peptide were considered background and the negative control, respectively. The experiments were repeated three times. The experiments were repeated three times.

If a protein-target interaction has a low affinity which results in a low phage ELISA signal, the affinity can be increased based on the published variants with a higher affinity. For instance, it was documented that the Cbx1 double mutant (K43A/D59F; Albanese et al., 2020) had a four-fold higher affinity, and the triple mutant (V22E/K25E/D59S; Hard et al., 2018) had ten-fold high affinity compared to the wild type Cbx1. These two mutants may have higher ELISA signals than the wild type. To test it, we mutated the fused Cbx1 to both variants using the Kunkel method, respectively, and measured their binding to H3K9me3 and H3K9 peptides by supernatant phage ELISA. Figure 3 showed that both mutants had around seven-fold stronger ELISA signals (0.38 or 0.53) for binding to the H3K9me3 peptide than those (~0.06) for binding to the H3K9 peptide. It indicates that the fused Cbx1 is well expressed. The advantage of this method is that even though the wild-type protein has a low affinity, it can be mutated as the variants with high affinity, which can be employed to check the expression of the variants on the surface. Therefore, the variants could be used as the library template. Its disadvantage is that such mutant may not have been reported.

### Approach Three: Combinatorial Binding Increases the ELISA Signals

**Figure 4:**
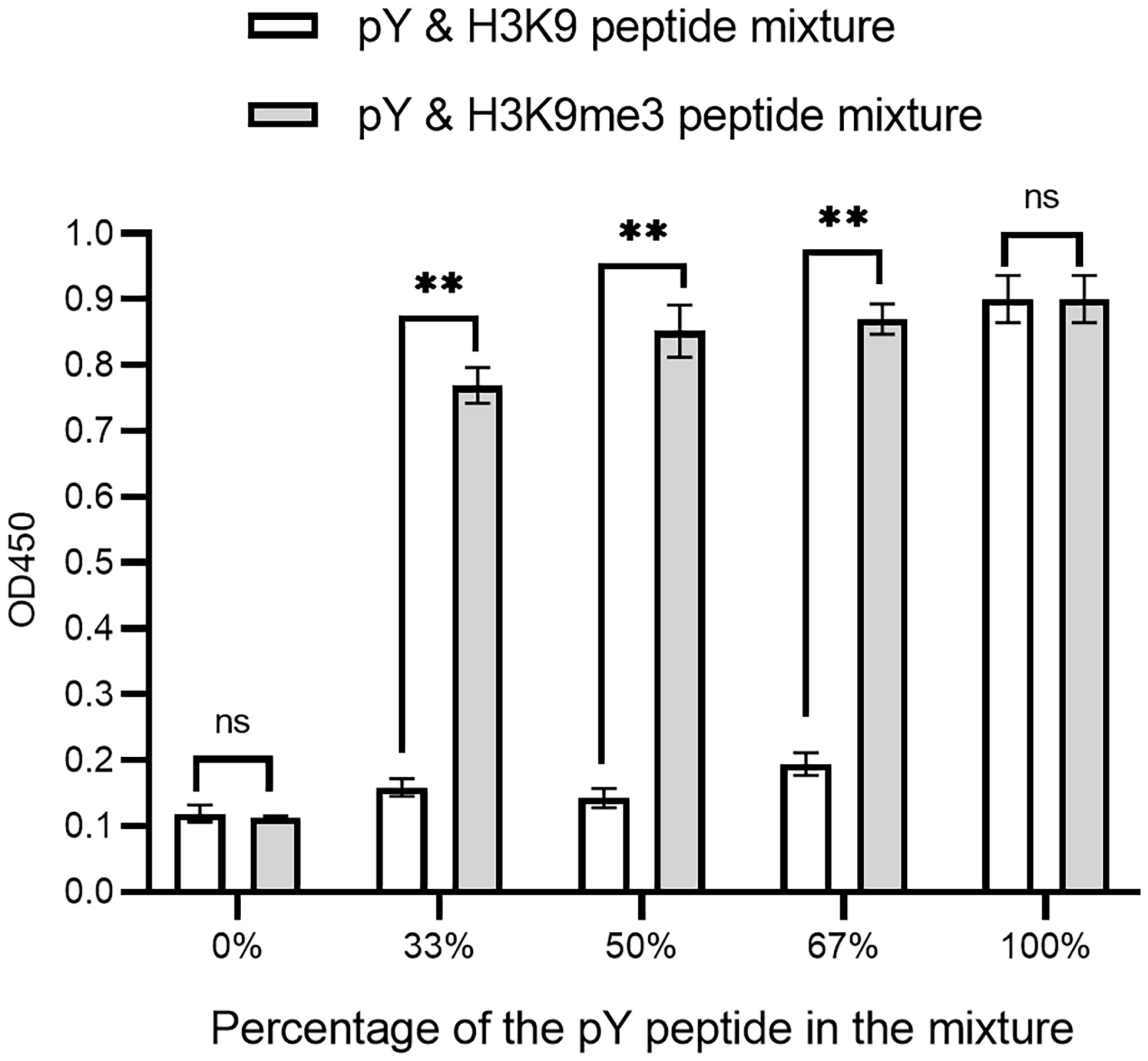
Supernatant ELISA signals were plotted as a function of the Cbx1-and-FynSH2-containing phage binding to the mixtures of pY and H3K9me3 peptides with different ratios. The proportion of pY peptide in the mixtures ranged from 0 to 100%. The mixtures of pY and H3K9 peptides were taken as the negative control. The experiments were repeated three times.

The affinity clamping technology has been developed by which a low-affinity peptide-binding domain is combined with a second domain to enhance affinity (Hard et al., 2018) dramatically. For example, a fibronectin type III domain (FN3) was attached to Grb2 SH2 so that a target peptide can be “clamped” with the improved affinity (Yasui et al., 2014). Inspired by this technology, we investigated whether Cbx1 is functionally displayed by joining it with the Fyn SH2 domain. We reasoned that binding affinity for the domain combination to the mixture of pY and H3K9me3 peptides is stronger than that of the mixture of pY and unmodified peptides. Accordingly, we fused both Fyn SH2 and Cbx1 to the capsid protein P3 and examined their binding to the immobilized mixture of pY and H3K9me3 peptides in different ratios, compared with the mixture of pY and the unmodified peptides in the same ratios. Figure 4 showed that supernatant phage ELISA signals (0.90) were solid and similar between the Cbx1-and-FynSH2-containing phage and the FynSH2-containing phage binding to the pY peptides, whereas the signals (0.11) were weak for both phages binding to the H3K9me3 peptides. It indicates that the Fyn SH2 domain is displayed on the phage surface. As the proportion of pY peptide in the modified peptide mixture decreased from 2/3 to 1/2 to 1/3, the ELISA signals were similar and high (0.77 ~ 0.87). By contrast, the signals significantly decreased from 0.91 to 0.12 with the proportion of the pY peptide in the mixture of pY and unmodified peptides (Figure 4). The significant differences of ELISA signals between two distinct peptide mixtures reveal that Cbx1 is functionally displayed on the phage surface. In the modified peptide mixture, pY and H3K9me3 peptides bind to the Cbx1-and-FynSH2-containing phage and the combinatorial binding increases the ELISA signals. By contrast, only the pY peptide contributes to the binding in another mixture, and the related ELISA signals are relatively low. In summary, it is feasible to examine the expression of a low-affinity protein through the assistance of another protein-target interaction.

### Estimation of These Approaches for Measuring Protein Expression With Relatively Low Affinity

**Figure 5:**
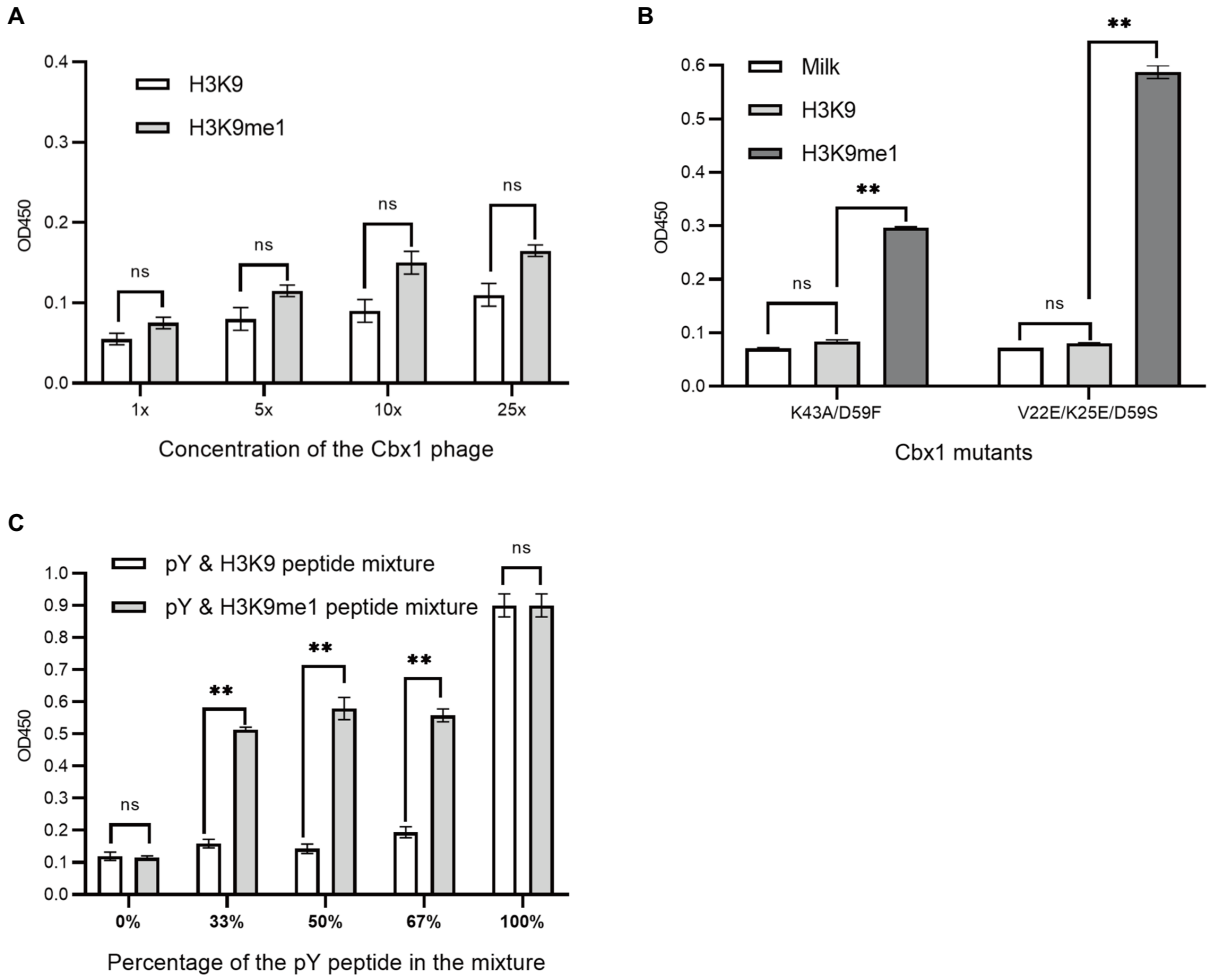
Phage ELISA showed the binding to the H3K9me1 peptide of the Cbx1 protein with different phage concentrations **(A)** or supernatant Cbx1-mutant-containing phages **(B)** or the binding of Cbx1-and-FynSH2-containing phage **(C)** to the mixtures of pY and H3K9me1 peptides with different ratios. The mixtures of pY and H3K9 peptides were considered as negative controls. The experiments were repeated three times.

We have introduced three approaches to determining whether Cbx1 can be well expressed on the phage surface through the ELISA test by testing its binding to the H3K9me3 peptide. We wondered if these methods could be applied to detect specific binding to a peptide with lower affinity than the Cbx1 and H3K9me3 complex. The binding of Cbx1 to the monomethyllysine peptide H3K9me1 has the K_d_ value (12.5 μM) four times larger than that (K_d_ = 3.2 μM) for the Cbx1 and H3K9me3 complex. We supposed that we only knew the Cbx1-H3K9me1 complex and used it to test the expression of the displayed Cbx1. We performed the binding of Cbx1 and the H3K9me3 peptide (Figure 2) and the H3K9me3 peptide (Figure 5A) simultaneously using phage ELISA. Figure 5A showed that high phage concentration failed to distinguish between the binding of Cbx1 to the H3K9me1 peptide and the H3K9 peptide, although the former had slightly stronger ELISA signals compared to the latter. Additionally, the approaches of protein mutation (Figure 5B) and combinatorial binding with the Fyn SH2 domain (Figure 5C) could increase the ELISA signals and show a significant difference compared with the negative control. These results suggest that the phage concentration approach may not be helpful to measure the proteins with much low binding affinity to their targets, whereas these proteins could be gauged using the mutation approach or the combinatorial binding approach.

## Discussion

Protein engineering can modulate the affinity of a protein to its target. Besides antibody engineering, natural modification-binding proteins are generally engineered to bind to pan-modified targets with enhanced affinity through surface display technologies. For example, the protein Af1521 was tailored to enrich the ADP-ribosylated proteome (Nowak et al., 2020), and the Fyn SH2 domain was engineered to enrich tyrosine phosphoproteome (Liu et al., 2019; Veggiani et al., 2019). As the natural protein has been identified to interact with the target with a certain affinity, their interaction can be used to measure whether the protein fused with the surface protein (e.g., phage P3, P8 or P9) is well and functionally expressed. Some of the protein-target complexes (e.g., the Fyn SH2 and MidT-pY324 complex) have relatively high affinity (e.g., K_d_ < 1 μM) so that the interactions are easily detected through supernatant phage ELISA. By contrast, others (e.g., the Cbx1 and H3K9me3 complex) have a poor affinity (e.g., K_d_ > 1 μM), and their weak interactions are facilely separated during elution and thus difficult to be identified using the simple supernatant phage ELISA approach. To solve this problem, we developed different approaches and compared them using, as the study case, the phage-displayed human Cbx1, which interacts with lysine methylation (Liu et al., 2010).

A straightforward approach is the increase of phage concentration. It indeed increases the ELISA signal but amplifies the background signal too. Therefore, this method may be suitable for detecting interactions with a medium affinity but not a weak affinity. Second, if the protein of interest has been reported to have the variant with enhanced affinity, the variant can be tethered to the surface protein, and its binding to the target can be tested using supernatant phage ELISA or concentrated phage ELISA. If the above two methods fail, we suggested fusing a small protein (or a peptide) with a known high affinity to its target to the phage-displayed protein and testing their binding to the mixture of their targets using ELISA. The rationale is that the affinity for the combined proteins binding to the mixture of both targets is stronger than that to the mixture of the target of the small protein and one non-target molecule because the former mixture has a greater valency to the latter mixture. Indeed, the Cbx1-and-FynSH2-containing phages showed higher ELISA signals when binding to the pY and H3K9me3 peptide mixture than binding to the pY and unmodified peptide mixture. The limitation of this method is that the length of the combined proteins should be considered as large proteins may be inefficiently displayed. The Fyn SH2 domain comprises approximately 100 amino acids, affecting its practical application. The Fyn SH2 domain and pY complex may be replaced by the complex of a short peptide and a protein with a similar affinity. For instance, the Fyn SH3 domain and the class I peptide (VSLARRPLPPLP) have a similar affinity [K_d_ = 0.18 μM (Ben-David et al., 2019)] to that (K_d_ = 0.327 μM) of the Fyn SH2 domain and MidT-pY324 peptide. Therefore, the fused SH2 domain may be replaced by the class I peptide and, accordingly, the pY peptide could be substituted by the Fyn SH3 domain.

## Data Availability Statement

The original contributions presented in the study are included in the article/supplementary material, further inquiries can be directed to the corresponding authors.

## Author Contributions

LL and RL supervised the whole project. CM, DZ, CJ, and SL designed the experiments. CM created the figures and tables. LL and CM wrote the manuscript. All authors contributed to the article and approved the submitted version.

## Funding

This work was supported by funds from the National Natural Science Foundation of China (Grant Nos. 31770821 and 32071430 to LL).

## Conflict of Interest

The authors declare that the research was conducted in the absence of any commercial or financial relationships that could be construed as a potential conflict of interest.

## Publisher’s Note

All claims expressed in this article are solely those of the authors and do not necessarily represent those of their affiliated organizations, or those of the publisher, the editors and the reviewers. Any product that may be evaluated in this article, or claim that may be made by its manufacturer, is not guaranteed or endorsed by the publisher.

## Abbreviations

ELISA: Enzyme-Linked ImmunoSorbent Assay
SH2: Src Homology 2
